# A modified renal risk score for Chinese patients with antineutrophil cytoplasmic antibody-associated vasculitis

**DOI:** 10.1186/s12916-023-02755-4

**Published:** 2023-02-08

**Authors:** Rui-Xue Wang, Jin-Wei Wang, Zhi-Ying Li, Su-Fang Chen, Xiao-Juan Yu, Su-Xia Wang, Fan Zhang, Zu-Ying Xiong, Shu-Hong Bi, Yue Wang, Ming-Hui Zhao, Min Chen

**Affiliations:** 1 Renal Division, Department of Medicine, Peking University First Hospital, Peking University Institute of Nephrology, Key Laboratory of Renal Disease, Ministry of Health of China, Key Laboratory of Chronic Kidney Disease Prevention and Treatment (Peking University), Ministry of Education, Research Units of Diagnosis and Treatment of Immune-mediated Kidney Diseases, Chinese Academy of Medical Sciences, Beijing, 100034 China; 2 Laboratory of Electron Microscopy, Pathological Centre, Peking University First Hospital, Beijing, 100034 China; 3 Renal Division, Department of Medicine, Peking University Shenzhen Hospital, Shenzhen, 518036 China; 4 Shenzhen Peking University-The Hong Kong University of Science and Technology Medical Center, Shenzhen, 518036 China; 5 Division of Nephrology, Peking University Third Hospital, Beijing, 100191 China; 6 Peking-Tsinghua Center for Life Sciences, Beijing, 100034 China

**Keywords:** ANCA, Vasculitis, Renal risk score, End-stage renal disease

## Abstract

**Background:**

The renal risk score (RRS) is a useful tool to predict end-stage renal disease (ESRD) in patients with antineutrophil cytoplasmic antibody (ANCA)-associated vasculitis (AAV). The current study aimed to validate the predictive performance of RRS and to further modify this model in Chinese AAV patients.

**Methods:**

Two hundred and seventy-two patients diagnosed with AAV confirmed by renal biopsies were retrospectively enrolled from a single center. The RRS was calculated based on 3 categorical variables, i.e., the proportion of normal glomeruli, the proportion of interstitial fibrosis and tubular atrophy (IF/TA), and eGFR at biopsy, classifying these patients into low-, medium-, and high-risk groups. In addition, a modified model was developed based on the RRS and was further validated in another independent cohort of 117 AAV patients. The predictive performance of each model was evaluated according to discrimination and calibration.

**Results:**

Patients were classified by the RRS into low- (26.5%), medium- (46.7%), and high-risk (26.8%) groups, with 120-month renal survival rates of 93.3%, 57.2%, and 18.4%, respectively (*P* < 0.001). The RRS showed good discrimination but less satisfactory calibration. Therefore, a modified model with improved discrimination and calibration was developed in Chinese AAV patients, with eGFR, proportion of normal glomeruli (both as continuous variables), and IF/TA (< 25%, 25–50%, > 50%) included. Internal and external validation of the modified model were performed. Finally, an online risk prediction tool was developed based on the modified model.

**Conclusions:**

The RRS was an independent predictor of ESRD of AAV patients. The modified model could predict the probability of ESRD for AAV patients with improved performance in Chinese AAV patients.

**Supplementary Information:**

The online version contains supplementary material available at 10.1186/s12916-023-02755-4.

## Background

Antineutrophil cytoplasmic antibody (ANCA)-associated vasculitis (AAV) comprises a group of autoimmune disorders, including microscopic polyangiitis (MPA), granulomatosis with polyangiitis (GPA), and eosinophilic granulomatosis with polyangiitis (EGPA), characterized by necrotizing small-vessel vasculitis with serum autoantibodies mainly against proteinase 3 (PR3) or myeloperoxidase (MPO). As one of the most common manifestations of AAV, ANCA-associated glomerulonephritis (ANCA-GN) presents in 80–100% of patients with MPA and 38–70% with GPA, which is typically characterized by pauci-immune necrotizing crescentic glomerulonephritis in renal histology [1–3].

Left untreated, AAV is a life-threatening disease. Immunosuppressive therapy, in particular, corticosteroids in combination with cyclophosphamide or rituximab, has dramatically improved the outcome of AAV patients, but a significant proportion of patients still progress to end-stage renal disease (ESRD). In a recent study with a training cohort of 115 patients and a validation cohort of 90 patients, a renal risk score (RRS) was developed by Brix et al. to predict renal outcome for AAV patients [4]. The RRS was based on a Cox model with clinical and pathological parameters at diagnosis, including proportion of normal glomeruli (N0 > 25%, N1 10–25%, N2 < 10%), proportion of interstitial fibrosis and tubular atrophy (IF/TA) (T0 ≤ 25%, T1 > 25%), and estimated glomerular filtration rate (eGFR) (G0 > 15 ml/min per 1.73 m^2^, G1 ≤ 15 ml/min per 1.73 m^2^). The RRS was calculated as the sum of the assigned points for each parameter (N1 = 4, N2 = 6, T1 = 2, G1 = 3 points). This score was designed to predict ESRD risk as low (0 points), medium (2 to 7 points), or high (8 to 11 points), and it proved to be a clinically applicable tool for early risk prediction of ESRD in ANCA-GN. Validation of the RRS was performed in 11 cohorts worldwide, comprising 37 to 252 patients, and showed its predictive value [5–15]. However, regarding discrimination and calibration, the two key aspects of a systematic assessment of predictive performance for a model, most of the previous validation studies did not report discrimination [5, 6, 8, 9, 12–15], and none of them reported calibration [5–15].

In the current study, a systematic validation was performed in Chinese AAV patients, which showed that the RRS was an independent predictor for ESRD with good discrimination but less satisfactory calibration. Therefore, model modification was launched to improve the predictive performance (including discrimination and calibration) of the RRS. Furthermore, internal and external validations were performed to assess the extent of optimism and overfitting of the modified model.

## Methods

### Patients

Two hundred and seventy-two patients with biopsy-confirmed ANCA-GN diagnosed in Peking University First Hospital from 1998 to 2019 and followed up for at least 12 months were retrospectively recruited. For the external validation cohort of the modified model, 117 patients with biopsy-confirmed ANCA-GN diagnosed from 2009 to 2021 were recruited from Peking University Third Hospital and Peking University Shenzhen Hospital. All patients met the Chapel Hill Consensus Conference nomenclature for AAV [16]. Patients with secondary vasculitis or other coexisting renal diseases were excluded. Patients with EGPA were excluded because compared with MPA and GPA, EGPA was increasingly recognized as a distinct type of AAV with different manifestations and outcomes [17]. Patients with ESRD at diagnosis were excluded as well. For eGFR calculation, Chronic Kidney Disease Epidemiology Collaboration (CKD-EPI) equation has been widely used in recent years, while in the original study of RRS by Brix et al. [4], eGFR was calculated with Modification of Diet in Renal Disease (MDRD) formula. So, in the current study, the eGFR was calculated according to both the MDRD equation for Chinese individuals [18] and the CKD-EPI equation [19]. The disease activity of AAV was assessed according to the Birmingham Vasculitis Activity Score (BVAS) [20].

This research was in accordance with the Declaration of Helsinki and was approved by the Ethics Committee of Peking University First Hospital (No.2019yan217), the Ethics Committee of Peking University Third Hospital (No.2022-171-01), and the Ethics Committee of Peking University Shenzhen Hospital (No.2020-044). Informed consent was signed by patients or their guardians.

### Renal histopathology evaluations

Renal histopathological evaluations were carried out by 2 independent pathologists, both of whom were blinded to the patients’ information. When differences in the same biopsy occurred, the biopsy was re-reviewed by the 2 pathologists until consensus was achieved. For adequate evaluation, biopsies with fewer than 10 total glomeruli were not included.

According to previous studies, the standardized definitions of renal pathological lesions were as follows [21–26]. In brief, normal glomeruli were defined as glomeruli without vasculitic lesions or sclerosis. Glomeruli with subtle changes because of ischemia or a minimum number of inflammatory cells were also regarded as normal glomeruli. As a mixture of cells, fibrin, and fibrous matrix, crescents referred to extracapillary lesions involving 10% or more of the circumference of Bowman’s capsule. Cellular crescents referred to glomeruli with cellular components more than 10% of the crescents. When the crescents were composed of more than 90% extracellular matrix, they were defined as fibrous crescents. Global glomerulosclerosis referred to glomeruli consisting of more than 80% sclerotic changes of the tuft. IF/TA was scored semiquantitatively as < 25%, 25–50% and > 50% according to the proportion of the affected tubulointerstitial compartment.

### Renal risk score

According to Brix’s study, the RRS was based on 3 parameters with predictive cutoff values [4]: normal glomeruli (N0 > 25%, N1 10–25%, and N2 < 10%), IF/TA (T0 ≤ 25% and T1 > 25%), and eGFR (G0 > 15 ml/min per 1.73 m^2^; G1 ≤ 15 ml/min per 1.73 m^2^). Furthermore, each degree of the parameters was assigned the same score point as the primary study by Brix et al. [4] (N1 = 4, N2 = 6, T1 = 2, G1 = 3 points), which were summed to attain the risk score. Finally, based on the risk score, three risk groups were defined as follows: low, 0 points; medium, 2–7 points; high, 8–11 points.

### Outcomes

The primary outcome of the current study was progression to ESRD, defined as the need for maintenance dialysis or kidney transplantation. The renal survival time for each patient was calculated from the diagnosis of AAV to ESRD or to the last follow-up or death.

### Statistical analyses

Data analyses were performed with SPSS (version 22.0, IBM Corp, Armonk, NY) and SAS (version 9.4, SAS Institute Inc., Cary, NC). Data were shown as the mean ± SD (for data that were normally distributed) or median and interquartile range (IQR; for data that were in skewed distribution) for continuous variables and number (%) for qualitative variables. The Mann-Whitney *U* test or crosstabs were performed to compare the baseline data of the training cohort and validation cohort. Univariable and multivariable Cox regression were performed as appropriate. Skewed-distributed data were converted into natural-logarithm (ln) form in Cox regression analyses. Multiple imputation was performed in the case of missing data. Kaplan-Meier analysis was employed to assess renal survival. *P* < 0.05 was considered statistically significant. The predictive performance of the models was assessed by discrimination (Harrell’s *C*-statistic) and calibration. Discrimination level ranged from 0.5 to 1.0 and approached 1.0 in the case of a perfect match. Calibration of the models was assessed by the Hosmer and Lemeshow test, in which *P* > 0.05 indicated no significant difference between the predicted probability estimated by the model and observed outcome frequencies during a certain period of time. Internal validation was performed using the one-shot method and presented as the 95% confidence interval (CI) of Harrell’s *C*-statistic [27]. In order to improve the performance of the original RRS in the setting of our cohort and maintain the clinical information suggested in Brix’s study [4], model modification was performed by re-estimation of regression coefficients of all predictors in the primary model as previously described by Moons et al. [28]. Since categorizing a continuous variable would cause loss of information, eGFR and the proportion of normal glomeruli were employed as continuous variates and IF/TA was still included as a categorical variate.

## Results

### General data of the training cohort

In the current study, 272 patients with ANCA-GN were recruited for the training cohort, which was an external validation cohort for the RRS as well. They were followed up for a median duration of 54.5 (IQR 32.0–89.0, range 12.0–246.0) months. Among these 272 patients, 125 were male, and 147 were female, with a median age of 61.0 (IQR 51.0–68.0, range 17.0–83.0) years at renal biopsy. Two hundred and fourteen (78.7%) patients were classified as MPA, while 58 (21.3%) patients were classified as GPA. MPO-ANCA and PR3-ANCA were positive in 246 (90.4%) and 26 (9.6%) patients, respectively. The median levels of serum creatinine and eGFR (calculated with MDRD equation for Chinese) were 288.5 (IQR 175.2–556.0) μmol/L and 16.9 (IQR 7.6–31.8) ml/min per 1.73m^2^ at renal biopsy, respectively. Eighty-two patients progressed to ESRD during a median follow-up of 21.5 (IQR 3.0–40.0, range 2.0–115.0) months. In the 79 (29.0%) patients with newly started dialysis at diagnosis, 50 patients had renal function restoration after immunosuppressive therapy, while 29 patients remained dialysis-dependent. Multiple imputation was performed in the case of missing data. The baseline data of the patients were presented in Table 1.

**Table 1:** General data and outcomes of the training and validation cohorts

Parameters	Training cohort (*n* = 272)	Validation Cohort (*n* = 117)	*P*
Age (years), median (IQR)	61.0 (51.0–68.0)	61.0 (56.0–66.5)	0.693
Gender			0.849
Male subjects, *n* (%)	125 (46.0)	55 (47.0)	
Female subjects, *n* (%)	147 (54.0)	62 (53.0)	
ANCA subtypes			0.555
MPO-ANCA, *n* (%)	246 (90.4)	108 (92.3)	
PR3-ANCA, *n* (%)	26 (9.6)	9 (7.7)	
Hb, g/dL, mean ± SD	9.6 ± 2.1^a^	9.16 ± 1.9	0.037
PLT, × 10^9^/L, median (IQR)	229.0 (178.3–304.8) ^a^	242.0 (180.3–307.0)	0.790
eGFR (ml/min per 1.73m^2^), median (IQR)	16.9 (7.6–31.8)	14.4 (8.5–31.1)	0.859
Urinary protein, g/24 h, median (IQR)	1.4 (0.6–2.4)^a^	1.6 (0.6–2.5)	0.615
BVAS, median (IQR)	17 (14–22)	17 (15–26)	0.207
Histological data			
Glomeruli, median (IQR)	26.0 (19.0–36.0)	28.0 (18.0–37.0)	0.800
Normal glomeruli (%), median (IQR)	25.0 (10.6–46.6)	25.0 (10.7–54.6)	0.860
Cellular crescents (%), mean ± SD	42.5 ± 22.8	41.5 ± 25.3	0.722
IF/TA			< 0.001
< 25%, *n* (%)	153 (56.2)	36 (30.8)	
25–50%, *n* (%)	85 (31.3)	43 (36.7)	
> 50%, *n* (%)	34 (12.5)	38 (32.5)	
C3 deposition > 1+, *n* (%)	88 (32.4)	41 (35.0)	0.717
Treatment			
Corticosteroids combined with CTX or RTX, *n* (%)	246 (90.4)	108 (92.3)	0.555
IV methylprednisolone pulse, *n* (%)	184 (67.6)	76 (65.0)	0.605
Plasma exchange, *n* (%)	58 (21.3)	27 (23.1)	0.701
Renal risk score, median (IQR)	5.0 (0.0–8.0)	5.0 (2.0–9.0)	0.142
Risk group			0.06
Low, *n* (%)	72 (26.5)	19 (16.2)	
Medium, *n* (%)	127 (46.7)	57 (48.7)	
High, *n* (%)	73 (26.8)	41 (35.0)	
ESRD, *n*	82	33	0.486

*Abbreviations*: *ANCA* anti-neutrophil cytoplasmic antibodies, *BVAS* Birmingham Vasculitis Activity Score, *C3* complement 3, *CTX* cyclophosphamide, *eGFR* estimated glomerular filtration rate, *ESRD* end-stage renal disease, *GPA* granulomatosis with polyangiitis, *Hb* hemoglobin, *IF/TA* interstitial fibrosis and tubular atrophy, *IQR* interquartile range, *IV* intravenous, *MPA* microscopic polyangiitis, *MPO* myeloperoxidase, *PLT* platelet, *PR3* proteinase 3, *RTX* rituximab, *Scr* serum creatinine

^a^Multiple imputation was performed in the case of missing data, including hemoglobin (*n* = 4), platelets (*n* = 8) and urinary protein (*n* = 20)

### Histological features of the training cohort

Patients had a median of 26 glomeruli (IQR 19–36) per biopsy. The proportions of normal glomeruli and globally sclerotic glomeruli were 25.0% (IQR 10.6–46.6%) and 14.8% (IQR 4.4–29.8%), respectively. Cellular crescents accounted for 42.5% ± 22.8% of the total glomeruli, while fibrous crescents accounted for 14.3% (IQR 4.5–29.5%) of the total glomeruli. There were 153 (56.2%), 85 (31.3%), and 34 (12.5%) patients with IF/TA< 25%, 25–50%, and > 50%, respectively.

### Validation of the RRS in the training cohort

**Fig. 1:**
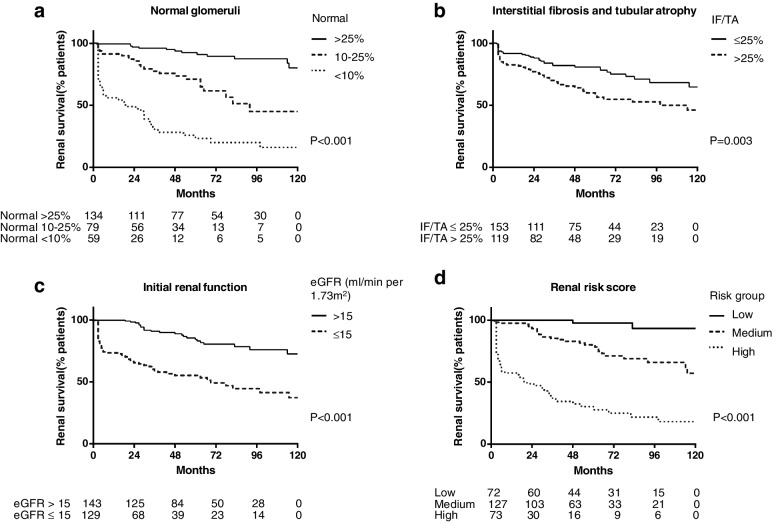
Renal survival curves for the primary model. Kaplan–Meier curves for **a** the proportion of normal glomeruli, **b** the area of IF/TA, **c** the eGFR at diagnosis, and **d** the renal risk score

The RRS was calculated in accordance with the previous study by Brix et al. [4]. The eGFR, the proportion of normal glomeruli, and IF/TA were included in this scoring system. All these three factors were significantly associated with renal outcome in univariable analyses. Specifically, Fig. 1 (a, b, c) demonstrated the discriminatory effect of each parameter in the RRS on renal survival.

The median RRS in the training cohort was 5.0 (IQR 0.0–8.0, range 0.0–11.0). Seventy-two (26.5%), 127 (46.7%), and 73 (26.8%) patients were classified into low-, medium-, and high-risk groups, respectively. During the 120-month follow-up, 2, 29, and 51 patients in the low-, medium-, and high-risk groups progressed to ESRD, respectively. The renal survival of patients in each group was shown in Fig. 1d. The renal survival rates at 36-month follow-up were 100%, 85.5%, and 36.5% for the low-, medium-, and high-risk groups, respectively (*P* < 0.001), with 60-month renal survival rates of 97.8%, 80.2%, and 28.0% for the three groups, respectively (*P* < 0.001) and 120-month renal survival rates of 93.3%, 57.2%, and 18.4%, respectively (*P* < 0.001). As a continuous variable, the RRS could predict renal survival in the univariable analysis (HR 1.36, 95% CI 1.27–1.46, *P* < 0.001).

The predictive value of the RRS was further evaluated in a multivariable Cox analysis. First, univariable Cox regression was performed to select potential risk factors for ESRD. The clinical parameters that showed discriminatory power on renal survival included age, eGFR, hemoglobin, platelet count in peripheral blood, and urinary protein at diagnosis. Regarding renal pathological parameters, in addition to the proportion of normal glomeruli and IF/TA as mentioned above, the proportion of glomeruli with cellular crescents, that with fibrous crescents, and that with global sclerosis, as well as C3 deposition, also showed a significant association with renal survival. Then, in the multivariable Cox regression model, the difference among the three risk groups was still significant after adjusting for other candidate predictive factors (*P* < 0.001) (Table 2, model 1). Moreover, as a continuous variable, the RRS was an independent predictor for ESRD in multivariable Cox analysis after adjusting for the abovementioned factors (HR 1.30, 95% CI 1.20–1.40, *P* < 0.001) (Table 2, model 2).

**Table 2:** Multivariable Cox analyses for ESRD

Parameters	Model 1 (*n* = 272)			Model 2 (*n* = 272)		
	HR	95% CI	*P*	HR	95% CI	*P*
Age (per 10 years)	0.80	0.67–0.96	0.016	0.78	0.65–0.94	0.008
Sex (male subjects vs. female subjects)	1.36	0.86–2.14	0.187	1.28	0.81–2.02	0.284
Urinary protein (g/24 h, expressed as ln)	1.10	0.84–1.44	0.502	1.17	0.89–1.53	0.268
Hb (g/dL)	0.88	0.78–1.00	0.050	0.88	0.79–1.00	0.041
PLT (× 10^9^/L)	0.98	0.95–1.00	0.031	0.98	0.95–1.00	0.028
C3 deposition (> 1+ vs. ≤ 1+)	1.31	0.82–2.11	0.259	1.20	0.75–1.91	0.454
RRS	----	----	< 0.001	1.30	1.20–1.40	< 0.001
High	Ref.	----	----	----	----	----
Medium	0.27	0.16–0.46	< 0.001	----	----	----
Low	0.03	0.01-0.15	< 0.001	----	----	----

*Abbreviations*: *CI* confidence interval, *C3* complement 3, *ESRD* end-stage renal disease, *Hb* hemoglobin, *HR* hazard ratio, *ln* natural logarithm, *PLT* platelet, *RRS* renal risk score

The predictive performance of the primary model by Brix et al. [4] was further assessed based on discrimination and calibration in this training cohort, which was an external validation cohort for the RRS as well. Harrell’s *C*-statistic for renal survival at 36 months was 0.832 and 0.855 in the training cohort (*n* = 115) and validation (*n* = 90) cohort of Brix’s study, respectively [4]. Harrell’s *C*-statistic calculated in the current training cohort (*n* = 272) according to Brix’s Cox regression model was 0.8545. However, the Hosmer and Lemeshow test showed less satisfactory calibration of the primary model (*P* < 0.0001, *g* = 5), which indicated that the predicted outcome by this model was not quite consistent with the observed outcome. In addition, there were a number of patients with preserved renal function after long-term follow-up in the high-risk group (36.5%, 28.0%, and 18.4% for 36, 60, and 120 months, respectively), while there were a number of patients progressing to ESRD in the medium-risk group (14.5%, 19.8%, and 42.8% for 36, 60, and 120 months, respectively), suggesting that obvious heterogeneity existed among patients within the same group. Therefore, model modification was launched to improve the predictive performance of the RRS.

### Model modification

**Fig. 2:**
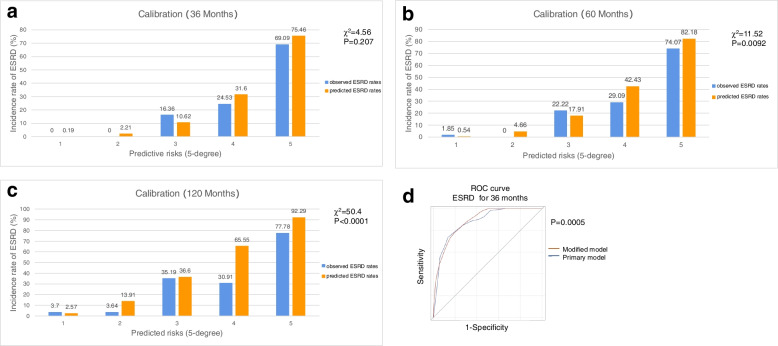
Calibration and discrimination of the modified model at 36 months (**a**), 60 months (**b**), and 120 months (**c**). Comparison of discrimination for the modified model and the primary model (**d**)

As shown in Table 3, a modified model was developed based on Brix’s model [4]. Similar to the primary model of Brix, eGFR (expressed as ln), proportion of normal glomeruli and IF/TA were included in the modified model, with the former two parameters as continuous variates and IF/TA as a categorical variate (< 25%, 25–50%, > 50%). In the modified model, Harrell’s *C*-statistics were 0.8936, 0.8786, and 0.8655 for renal survival at the follow-up of 36, 60, and 120 months, respectively. The Hosmer and Lemeshow test of the modified model showed *P* = 0.2070, *P* = 0.0092, and *P* < 0.0001 (*g* = 5) for renal survival of the follow-up of 36, 60, and 120 months, respectively, indicating no significant disagreement between model prediction and observed outcomes during 36-month follow-up (Fig. 2a, b, and c). Furthermore, we compared Harrell’s C-statistic of the modified model and Brix’s model in the current training cohort (*n* = 272). There was a significant difference in the C-statistic at the 36-month follow-up (0.8936 vs. 0.8545, *P* = 0.0005) (Fig. 2d). Re-analysis with eGFR calculated using CKD-EPI equation showed similar results with those of MDRD-calculated eGFR (Additional file 1: Figure S1).

**Table 3:** The modified model established by Cox regression (*n* = 272)

Parameters	36 months			60 months			120 months		
	HR	95% CI	*P*	HR	95% CI	*P*	HR	95% CI	*P*
eGFR (ml/min per 1.73 m^2^, expressed as ln)	0.37	0.25–0.56	< 0.001	0.45	0.31–0.65	< 0.001	0.55	0.40–0.77	< 0.001
IF/TA	----	----	0.056	----	----	0.012	----	----	0.036
IF/TA < 25%	Ref.	----	----	Ref.	----	----	Ref.	----	----
IF/TA 25–50%	1.40	0.77–2.55	0.268	1.60	0.91–2.80	0.100	1.26	0.76–2.09	0.374
IF/TA > 50%	2.34	1.17–4.68	0.016	2.66	1.39–5.08	0.003	2.17	1.20–3.93	0.010
Proportion of normal glomeruli	0.00	0.00–0.01	< 0.001	0.00	0.00–0.02	< 0.001	0.01	0.00–0.03	< 0.001

*Abbreviations*: *CI* confidence interval, *eGFR* estimated glomerular filtration rate, *HR* hazard ratio, *IF/TA* interstitial fibrosis and tubular atrophy, *ln* natural logarithm

### Internal validation of the modified model

Internal validation was performed for the modified model. Internal validation was commonly performed by randomly splitting the dataset into a training group and a validation group. However, this approach could “waste data” because not all available cases in the cohort were employed to develop a prediction model [29]. Additionally, it could result in “replication instability” in different random splits of the total cohort [29]. Therefore, in the current study, internal validation of our modified model was performed in the training cohort through the one-shot method, with the results presented as the 95% CI of Harrell’s *C*-statistic [27]. The study of Kang L et al. showed that the one-shot method compared favorably to the bootstrap method [27]. Harrell’s *C*-statistics were 0.8936 (95% CI 0.8595–0.9277), 0.8786 (95% CI 0.8433–0.9139), and 0.8655 (95% CI 0.8298–0.9012) for renal survival at the 36-, 60-, and 120-month follow-ups, respectively. The above data suggested that our modified model had good discrimination and performed well in internal validation.

### External validation of the modified model

For external validation of the modified model, an independent cohort of 117 patients with ANCA-GN was recruited from two other hospitals, i.e., Peking University Third Hospital and Peking University Shenzhen Hospital. The median follow-up period was 40 (IQR 22.5–62.5) months, and 33 patients developed ESRD during a median follow-up of 12 (IQR 3.0–25, range 1.0–79) months. Treatment protocols were similar between training and validation cohorts. The general data of these patients was also shown in Table 1. As for discrimination, Harrell’s *C*-statistic was 0.8681 for renal survival in the external validation cohort, which was comparable to that (0.8936) in the training cohort. The Hosmer and Lemeshow test of the modified model showed *P* = 0.1202 (*g* = 5) for renal survival in the external validation cohort, indicating no obvious disagreement between predicted and observed outcomes.

**Fig. 3:**
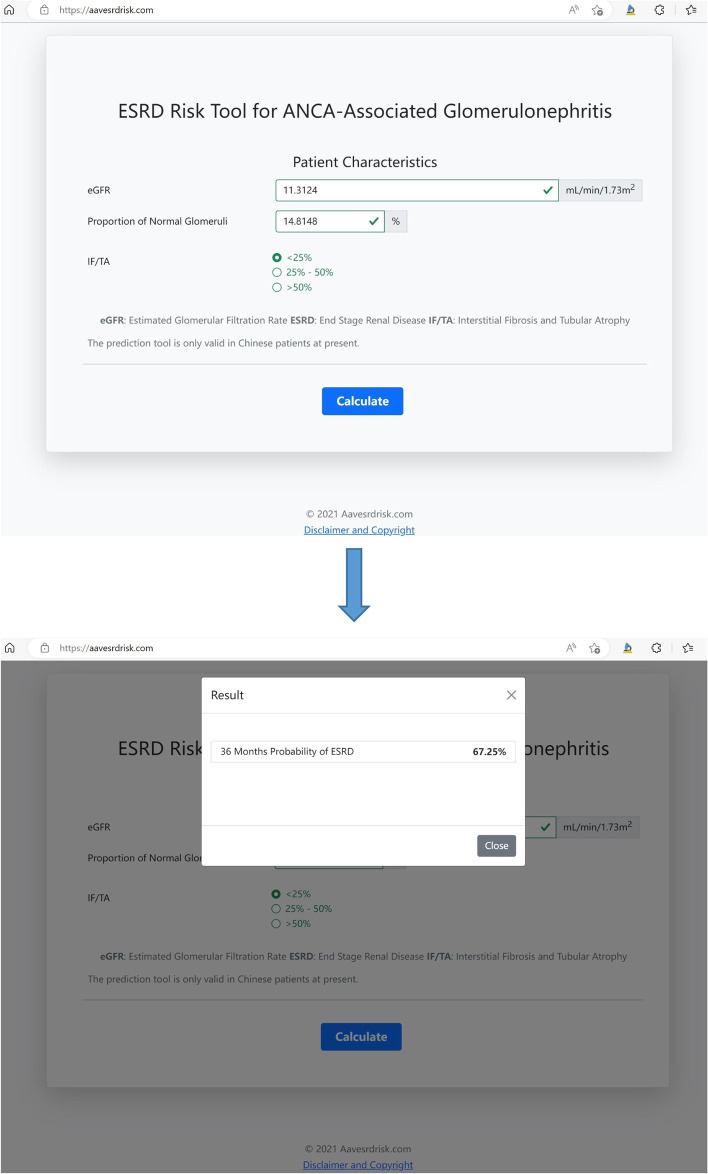
ESRD risk assessment via an online tool

For clinical use, an online risk prediction tool was developed based on the modified model [30], which can calculate the probability of an individual AAV patient progressing to ESRD in the percentage form (Fig. 3).

## Discussion

Renal involvement in AAV, namely, ANCA-GN, was associated with ESRD and poor outcome [31–33]. In China, AAV was an important cause of secondary glomerular diseases and the leading cause of acute kidney injury in elderly patients who received renal biopsy [34, 35]. Although the outcome of AAV has been dramatically improved by immunosuppressive therapy, a large number of patients still progressed to ESRD. Accordingly, it was of interest to establish and validate tools to predict renal outcome in patients with ANCA-GN.

The study of Berden et al. emphasized the significance of histological injury for renal outcome and demonstrated the ascending probability of progressing to ESRD from the focal to crescentic, mixed, and sclerotic categories [22]. However, such a sequence of the probability of progressing to ESRD was inconsistent among different studies, which indicated that the predictive significance of the classification may not be quite stable in different cohorts and its clinical application was limited to some extent [36, 37]. In addition, the histopathological classification was not predictive in the multivariable model involving eGFR, IF/TA and age [4, 38]. To establish a more reliable predictive model, Brix et al. developed and validated an RRS system to predict renal survival in German cohorts [4], and this model has been further validated in different cohorts worldwide [5–15]. However, the systematic assessment of predictive performance, including discrimination and calibration, was limited in these studies. Therefore, in the current study, a systematic evaluation of the predictive performance of the RRS in a Chinese cohort of AAV was launched.

Compared with previous studies for validation of RRS in ANCA-GN [5–15], the sample size of our study is the largest, with 272 patients. In addition, these 272 patients had a median of 26 (IQR, 19–36) glomeruli per biopsy, which was so-far the highest among these studies for the RRS, making the histopathological results more reliable and accurate. It was found in the current study that renal survival was significantly different among the high-, medium-, and low-risk groups, and Harrell’s *C*-statistic was satisfactory. Multivariate Cox analysis showed that the RRS was an independent predictor for ESRD after adjusting for other parameters, including age, sex, and urinary protein. Therefore, the RRS proved to be prognostic and practical in Chinese AAV patients and facilitated its clinical prognostication.

However, despite the satisfactory discrimination, the calibration assessment showed that the predicted outcomes by the RRS were not quite consistent with the observed outcomes (Hosmer and Lemeshow test, *P* < 0.0001). In addition, as mentioned above, obvious heterogeneity existed among the patients within the same group. Accordingly, based on Brix’s model, we further developed a modified model in Chinese AAV patients. Compared with the primary model, the modified model had improved discrimination and calibration for renal outcome. There were only 3 parameters in this model, i.e., proportion of normal glomeruli, IF/TA and eGFR, making it easily available in biopsy-proven ANCA-GN. The internal and external validation showed good discrimination and no significant disagreement between predicted and observed outcomes for a follow-up of 36 months. To facilitate clinical application, an online risk prediction tool based on the modified model was developed for physicians. Once the proportion of normal glomeruli, IF/TA and eGFR are entered, the website will quantify the probability of an individual AAV patient progressing to ESRD.

There were some limitations in our study. First, since this was a study in Chinese AAV cohorts with predominant MPA and MPO-ANCA patients, considering the heterogeneity of ANCA status in different populations, it might limit the extrapolation of this modified model. Validation of the modified model deserved further investigation in international cohorts. Second, patients in the training cohort were retrospectively recruited from a single center, and therefore, our modified model was not comparable with the original score designed from a multicenter prospective cohort. Third, the calibration was improved with the modified model at 36-month follow-up (Hosmer and Lemeshow test, *P* = 0.2070) but still showed a significant difference of prediction at 60-month (Hosmer and Lemeshow test, *P* = 0.0092) and 120-month follow-up (Hosmer and Lemeshow test, *P* < 0.0001). ESRD risk calculated by the modified model might be overestimated for high-risk patients in models of 60- and 120-month follow-up.

## Conclusions

In conclusion, a validation of the RRS was performed in our study, which demonstrated that the RRS was prognostic for ESRD but not fully satisfactory in Chinese AAV patients. Therefore, a modified model was established based on the RRS, with improved discrimination and calibration. Despite the not fully satisfying calibration of the modified model, this was an important investigation of the improvement for prediction of renal survival in Chinese AAV patients. Internal and external validation of the modified model showed good performance in Chinese AAV patients. An online risk tool derived from this model was developed, which may be practical to physicians.

## Supplementary Information

**Additional file 1: Figure S1.** Confirmation of the modified model with eGFR calculated using CKD-EPI equation. Calibration and discrimination of the modified model at 36 months (a), 60 months (b) and 120 months (c). Comparison of discrimination for the modified model and the primary model (d).

## Data Availability

The datasets used and/or analyzed during the current study are available from the corresponding author on reasonable request.

## Abbreviations

ANCA: Antineutrophil cytoplasmic antibody
AAV: Antineutrophil cytoplasmic antibody-associated vasculitis
MPA: Microscopic polyangiitis
GPA: Granulomatosis with polyangiitis
EGPA: Eosinophilic granulomatosis with polyangiitis
PR3: Proteinase 3
MPO: Myeloperoxidase
ANCA-GN: ANCA-associated glomerulonephritis
ESRD: End-stage renal disease
RRS: Renal risk score
IF/TA: Interstitial fibrosis and tubular atrophy
eGFR: Estimated glomerular filtration rate
CKD-EPI: Chronic Kidney Disease Epidemiology Collaboration
MDRD: Modification of Diet in Renal Disease
BVAS: Birmingham Vasculitis Activity Score
IQR: Interquartile range
CI: Confidence interval
