# Supplementary figures and images for: A modified renal risk score for Chinese patients with antineutrophil cytoplasmic antibody-associated vasculitis
Source: BMC Med. 2023 Feb 8;21:45. doi: 10.1186/s12916-023-02755-4 (PMC9909876; doi:10.1186/s12916-023-02755-4)

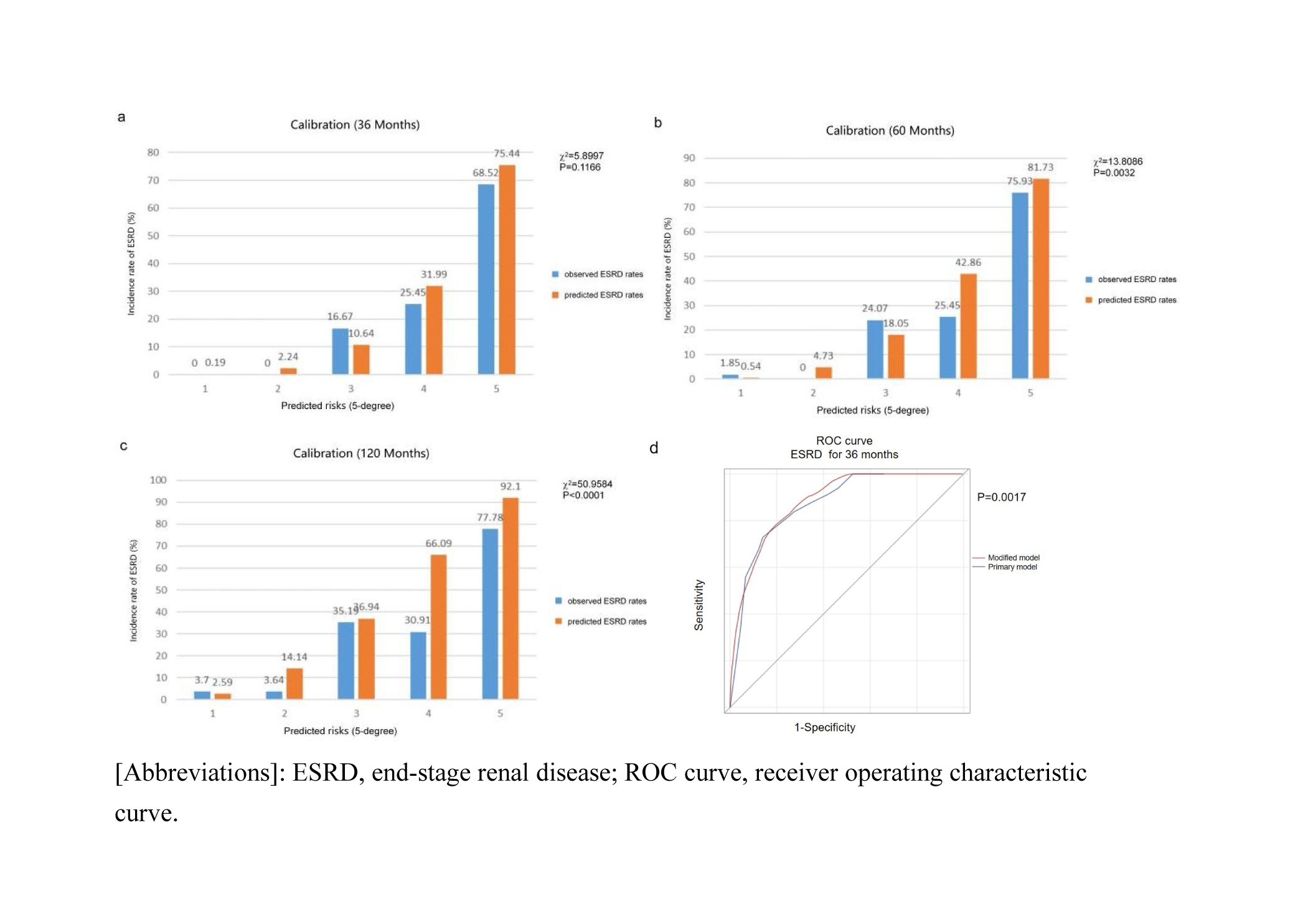

Supplement: Supplementary file 1 — Additional file 1: Figure S1. Confirmation of the modified model with eGFR calculated using CKD-EPI equation. Calibration and discrimination of the modified model at 36 months (a), 60 months (b) and 120 months (c). Comparison of discrimination for the modified model and the primary model (d). [file 12916_2023_2755_MOESM1_ESM.tif]
